# Ergonomic strategies to improve radiographers’ posture during mammography activities

**DOI:** 10.1007/s13244-017-0560-7

**Published:** 2017-06-21

**Authors:** Nicolai Cernean, Florentino Serranheira, Pedro Gonçalves, Cláudia Sá dos Reis

**Affiliations:** 1 Escola Superior de Tecnologia da Saúde de Lisboa/Instituto Politécnico de Lisboa, Lisboa, Portugal; 2 Escola Nacional de Saúde Pública, Universidade Nova de Lisboa, Lisboa, Portugal; 3 Centro de Investigação em Saúde Pública, Escola Nacional de Saúde Pública, Universidade Nova de Lisboa, Lisboa, Portugal; 4 Department of Medical Radiation Sciences, Curtin University, Perth, Western Australia

**Keywords:** Mammography, Radiographer, Ergonomics, Posture, Work-related disorders

## Abstract

**Objectives:**

To identify alternatives for radiographers’ postures while performing mammography that can contribute to reduce the risk of work-related musculoskeletal disorders (WRMSDs).

**Methods:**

Radiographers’ postures to positioning craniocaudal (CC) and mediolateral oblique (MLO) views were simulated without any intervention for three scenarios: radiographer/patient with similar statures, radiographer smaller than patient and radiographer taller than patient. Actions were taken to modify the postures: seated radiographer; patient on a step; seated patient; radiographer on a step. All the postures were analysed using kinovea 0.8.15 software and the angles were measured twice and classified according to European standard EN1005–4: 2005.

**Results:**

The non-acceptable angles were measured mainly during MLO positioning when radiographer was taller than the patient: 139° and 120° for arm-flexion and abduction, 72° for trunk and −24° for head/neck-flexion. The introduction of alternative postures (radiographer seated), allowed improvements in posture (60° and 99° for arm flexion and abduction, 14° for trunk and 0° for head/neck flexion), being classified as acceptable.

**Conclusions:**

The alternative postures simulated have the potential to reduce the risk of developing WRMSDs when radiographers and patients have different statures.

***Main messages*:**

• *Radiographers’ postures in mammography can contribute to work-related musculoskeletal disorders*

• *Non-acceptable posture was identified for MLO breast positioning (radiographer taller than patient)*

• *Adapting posture to patient biotype reduces the WRMSD risk for radiographers*

## Introduction

Mammography is the main imaging modality used in breast cancer screening and for that reason it is used frequently. To perform this exam, radiographers need to repeat movements and need to assume awkward postures to position the breast for standard views: craniocaudal (CC) and mediolateral oblique (MLO). The repetition of movements using extreme postures, the equipment manipulation requirements, the long working hours, heavier patient loads, less staff coverage, fewer opportunities for downtime and working in an environment with low temperatures can promote the development of work-related musculoskeletal disorders (WRMSDs) [1–3].

Regarding an occupational health perspective, WRMSDs should be prevented as opposed to treated because primary prevention is more effective than treatment [2, 4, 5].

Considering radiographers’ work scenarios, it is possible to identify several risk factors that should be evaluated (risk assessment) and mitigated (risk management) to prevent occupational diseases. The work environment and the equipment interface and its manipulation are highlighted as promoters of musculoskeletal discomfort during the patient positioning and examination by previous studies in the field of radiology [6–8]. Most of the studies are focused on the radiologists’ work, mainly in those situations related to interaction with the information systems, performing ultrasound exams, analysing and reporting exams [6–8]. Regarding radiographers’ work, very few references focused specifically on mammography activities were found in national or international contexts [1]. For that reason, the optimisation of radiographers’ work to prevent musculoskeletal symptoms and pain, as well as WRMSDs, is hard and not supported by strong evidence. In a mammography room, radiographers need to respond to multiple demands, adapting their attitudes and behaviours to the equipment layout and to the patient’s characteristics when performing the exam. In a recent study [1], problems related to postures inside mammography rooms were highlighted mainly when the radiographer and patient have different statures. The breast positioning for the acquisition of standard views—craniocaudal (CC) and mediolateral-oblique (MLO)—can be very demanding, requiring awkward postures classified as risk non-acceptable for WRMSDs. Designers and medical equipment designers, in Europe should consider patients’ and workers’ anthropometry characteristics to design interfaces (man-machine) to prevent WRMSDs (EN-1005-4: 2005).

The identification of strategies based on the ergonomic principals is essential to improve work interfaces related to equipment manoeuvring and patient positioning. Those strategies can contribute to preventing WRMSDs, reducing the health costs associated with healthcare professionals and patient safety [2, 4, 5, 7].

The aim of this study is to identify new strategies to improve the radiographer’s posture during the performance of mammography exams, to reduce the risk of WRMSD occurrence and to contribute to improving the quality of mammography exams.

## Methodology

The study was performed in two phases, using a mammography device from Siemens (Mammomat 1000; Siemens Medical Solutions, Erlangen, Germany) installed in the mammography laboratory at Lisbon School of Health Technology (ESTeSL).

In the first phase, the simulation of breast positioning in CC and MLO views using volunteers was carried out without any intervention in radiographer posture. Photographs and videos recorded were acquired (equipment Canon SX270 HS) in three different scenarios:

- The radiographer is taller than the patient (anthropometric stature radiographer/patient combination 180 cm/153 cm)
- The radiographer and patient have the same stature approximately (anthropometric stature radiographer/patient combination 171 cm/173 cm)
- The radiographer is smaller than the patient (anthropometric stature radiographer/patient combination 153 cm/173 cm)

Three observers visualised the video and the frames showing the most demanding postures were selected via consensus (1.103, 1.221 and 1.213) for the three scenarios previously presented [1]. Those frames were then introduced in specific software, kinovea 0.8.15, to measure the main body angles twice (head/neck, trunk and arms) according to the methodology proposed by Kapitaniac. The measured angles were classified in agreement with EN1005–4: 2005 [9, 10] in three different levels: “acceptable”, “conditionally acceptable” and “not acceptable” (Table 1).

**Table 1:** Reference values for postural assessment (Standard BS EN 1005–4: 2005)

Norma BSEN 1005–4: 2005 + A1: 2008			
Anatomical area/posture	Acceptable	Conditionally acceptable	Not acceptable
Trunk forward bending	0–20°	20–60°	>60°
Upper arm flexion	0–20°	20–60°	>60°
Upper arm abduction	0–20°	20–60°	
Head/neck upward/downward bending	−40 to 0°	-	0–40°

The European Standard used as reference in the study was selected because it specified the requirements for postures and movements at three levels with minimal external force. The requirements are intended to reduce the health risks for nearly all healthy adults.

In the second phase, alternative postures for the three scenarios were simulated using different strategies such as: (1) sit on a stool (height variable between 50 and 80 cm), (2) stand-up on a step (10 cm high) or (3) stand-up on another step (15 cm high), both 68.5 cm × 28 cm (Table 2). The simulations were also recorded (photographs and video) and the same methodology applied in the first phase was followed.

**Table 2:** Simulated contexts during the breast positioning in craniocaudal (CC) and mediolateral oblique (MLO), without and with postural alternatives considering the radiographer and the patient

Context	Postural alternatives for the radiographer	Postural alternatives for the patient	Simulated breast positioning
Anthropometric combination radiographer/ patient: 171 cm/173 cm (similar statures)	None	Patient on a *step* (10 cm)	CC
	None	Patient seated on a stool	CC
	None	Patient on a *step (10 cm)*	MLO
	Radiographer seated on a stool	None	MLO
Anthropometric combination radiographer/ patient: 180 cm/153 cm (radiographer taller than the patient)	None	Patient on a *step* (10 cm)	CC
	None	Patient on a *step* (15 cm)	MLO
	Radiographer seated on a stool	None	MLO
Anthropometric combination radiographer/ patient: 153 cm/173 cm (radiographer smaller than the patient)	Radiographer on a *step* (10 cm)	None	CC
	None	Patient seated on a stool	CC
	None	Patient on a *step* (10 cm)	MLO
	Radiographer seated on a stool	Patient seated on a stool	MLO

## Results

**Fig. 1:**
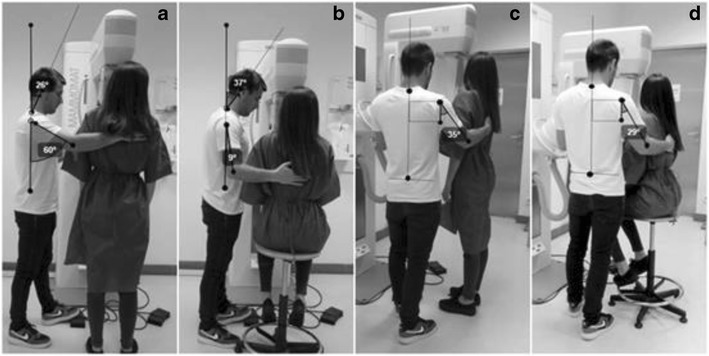
CC breast positioning: **a** and **c** radiographer postures without intervention; **b** and **d** alternative postures when the radiographer and patient have identical statures

**Fig. 2:**
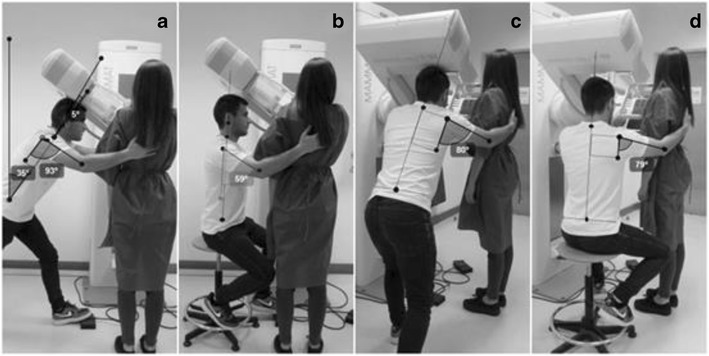
MLO breast positioning: **a** and **c** radiographer postures without intervention; **b** and **d** alternative postures when the radiographer and patient have identical statures

**Fig. 3:**
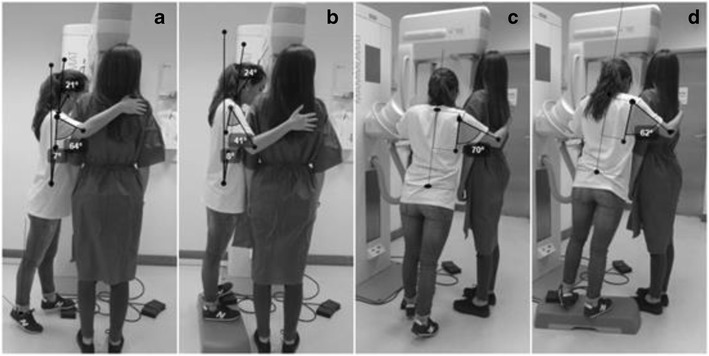
CC breast positioning: **a** and **c** radiographer postures without intervention; **b** and **d** alternative postures when the radiographer smaller than the patient

**Fig. 4:**
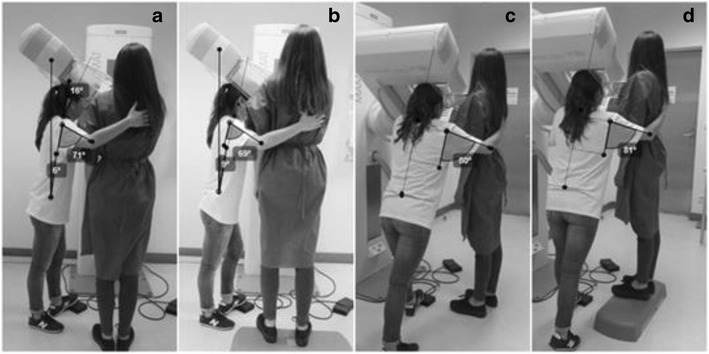
MLO breast positioning: **a** and **c** radiographer postures without intervention; **b** and **d** alternative postures when the radiographer smaller than the patient

**Fig. 5:**
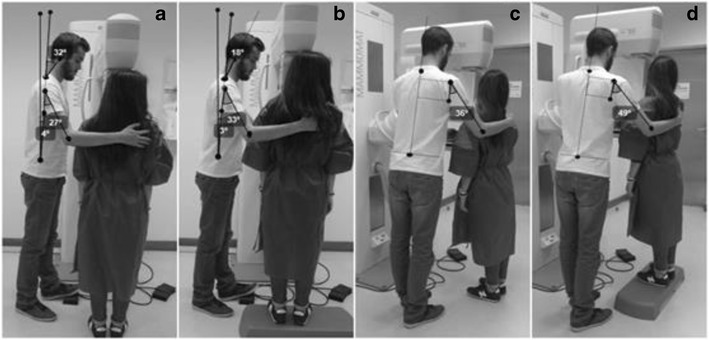
CC breast positioning: **a** and **c** radiographer postures without intervention; **b** and **d** alternative postures when the radiographer is taller than the patient

**Fig. 6:**
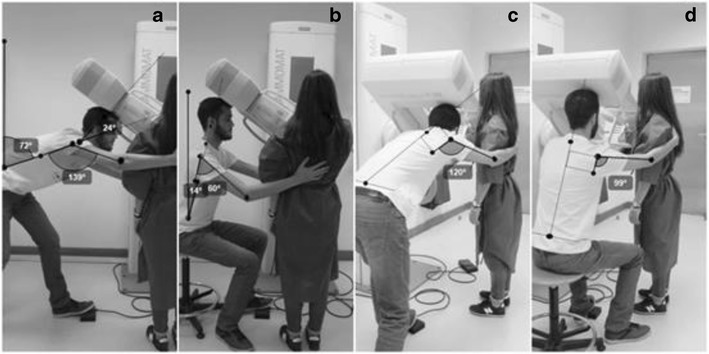
MLO breast positioning: **a** and **c** radiographer postures without intervention; **b** and **d** alternative postures when the radiographer is taller than the patient

The results are presented in three groups according to the three anthropomorphic scenarios simulated to positioning the breast in CC and MLO, without (Figs. 1, 2, 3, 4, 5 and 6a and c) and with interventions (Figs. 1, 2, 3, 4,5 and 6b and d).

### The radiographer and patient with identical stature (anthropometric stature radiographer/patient combination 171 cm/173 cm)

#### CC patient positioning

The radiographer assumed an orthostatic posture to positioning the breast for CC view. The trunk/spine was aligned according to the mid-sagittal plane. The right arm assumed a slight flexion and the forearm performed a rotation in the inner direction, allowing the palm of the hand to support the patient’s back. The left hand (not visible in the image) smoothed the breast down and forward with the fingers, helping the breast positioning and breast compression. The right leg supported part of the radiographer’s body weight, while the left leg performed a slight flexion to reach the compressor foot pedal. According to the European Norm, the posture was classified as “acceptable” considering the trunk and neck/head angles (Table 3). The right arm position was classified as “conditionally acceptable”.

**Table 3:** Angles measured on the radiographer during CC breast positioning (radiographer and patient with identical the stature)

		Without intervention		Patient on a *step*		Patient seated on a stool	
Posture	Breast positioning	Measured angle	Classification	Measured angle	Classification	Measured angle	Classification
Trunk	CC	0°	Acceptable	0°	Acceptable	0°	Acceptable
Arm flexion	CC	60°	Conditionally acceptable	38°	Conditionally acceptable	9°	Acceptable
Arm abduction	CC	35°	Conditionally acceptable	37°	Conditionally acceptable	29°	Conditionally acceptable
Head/neck	CC	26°	Acceptable	20°	Acceptable	37°	Acceptable

Observing the *postural alternatives*, namely the patient’s placement on a step, the angle of the arm in abduction was not reduced and the angle of the arm in flexion was still classified as “conditionally acceptable”.

Seating the patient to position the breast for CC view allowed an improvement of the arm angle during flexion and abduction, reducing the angles from 37 to 29° and from 38 to 9°, respectively (Table 3). With the angle reduction for the flexion movement, the classification was changed to “acceptable” according to the European standard.

#### MLO patient positioning

The MLO patient positioning required that the radiographer’s trunk and neck/head were in slight flexion, allowing observation of the breast to verify if all the tissue is included in the light field (that corresponds to the radiation beam field). The right arm remained flexed, and the hand helped to support the patient’s back. The right leg supported the radiographer’s body weight, while the left leg was positioned to easily reach the compression foot pedal (Fig. 2a). The angle of the trunk was considered “conditionally acceptable” measuring 35°. The angles measured with the arm flexed (93°) and abducted (80°) and the angles of the head/neck (−5°) were classified as “not acceptable” (Table 4).

**Table 4:** Angles measured on radiographer during MLO breast positioning (radiographer and patient with identical the stature)

		Without intervention		Patient on a *step*		Radiographer seated on a stool	
Posture	Breast positioning	Measured angle	Classification	Measured angle	Classification	Measured angle	Classification
Trunk	MLO	35°	Conditionally acceptable	26°	Conditionally acceptable	0°	Acceptable
Arm flexion	MLO	93°	Not acceptable	82°	Not acceptable	59°	Conditionally acceptable
Arm abduction	MLO	80°	Not acceptable	82°	Not acceptable	79°	Not acceptable
Neck/head	MLO	−5°	Not acceptable	−7°	Not acceptable	0°	Acceptable

The *postural alternatives* that were simulated with the patient being placed on a 10-cm high step (not displayed) allowed a reduction of 9° in the angle of trunk, changing the classification to “conditionally acceptable”. The changes in the angles of the arm abduction and head/neck were not very obvious (less than 3°).

The *alternative postures* promoted an improvement in the angles of the trunk and neck/head due to a reduction from 35 to 0°, being classified as “acceptable”. The flexion of right arm was improved and the position was changed from “not acceptable” (82°) to “conditionally acceptable” (59°). The abduction of the arm kept the classification as “not acceptable” when in alternative posture was applied (Table 4).

### Radiographer smaller than the patient—anthropometric combination 153 cm/173 cm

#### CC patient positioning

For the acquisition of a CC view, the radiographer assumed an orthostatic posture with the trunk/spine aligned with the mid-sagittal plane of body. Both arms were flexed and abducted. The right forearm rotated to the internal side, and the palm of the hand was supporting the patient’s back, ensuring that the patient kept the adequate position. The left hand was holding the patient’s breast, applying a slight pressure to help on the compression and ensuring that the nipple was positioned in profile as required by image quality criteria. In this specific situation, due to the height difference between the radiographer and the patient, observing the breast to verify if it was aligned and in the middle of light/radiation field was difficult. To observe those criteria, the radiographer needed to do an extension of the feet, leaning on the distal area (the metatarsal heads and toes) (Fig. 3a and c).

The radiographer’s posture without intervention was classified as “acceptable” considering the trunk and neck/head angulation, but classified as “not acceptable” for the arm flexion and abduction (Table 5).

**Table 5:** Angles measured on the radiographer during CC breast positioning (radiographer smaller than the patient)

		Without intervention		Patient seated in a stool		Radiographer on *step*	
Posture	Breast positioning	Measured angle	Classification	Measured angle	Classification	Measured angle	Classification
Trunk	CC	7°	Acceptable	7°	Acceptable	8°	Acceptable
Arm flexion	CC	64°	Not acceptable	36°	Conditionally acceptable	41°	Conditionally acceptable
Arm flexion	CC	70°	Not acceptable	62°	Not acceptable	42°	Conditionally acceptable
Head/neck	CC	21°	Acceptable	4°	Acceptable	24°	Acceptable

The *postural alternative* of seating the patient on a stool allowed the radiographer’s trunk to keep a posture considered as “acceptable” according European standards. However, improvements were noticed for the arm flexion and abduction. The angle of the flexed arm without intervention was classified as “not acceptable” (68°) changing to “conditionally acceptable” (36°) with the intervention (Table 5). The position of the trunk, head/neck of the radiographer stayed “acceptable”.

#### MLO positioning

The radiographer maintained a neutral position of the head/neck. The right arm was in flexion and abduction, and the palm of the hand was on the patient’s back. The right leg supported part of the radiographer’s body weight, while the left leg performed a slight flexion to reach the compressor foot pedal.

The trunk angle (6°) and head/neck angle (16°) were classified as “acceptable” without any intervention in radiographer’s posture (Fig. 4a and c). The arm angles in flexion (71°) and abduction (80°) were classified as “not acceptable” according the European standard (Table 6).

**Table 6:** MLO breast positioning: postures for radiographer smaller than the patient without and with postural interventions

		Without intervention		Radiographer and patient seated in a stool		Patient on a *step*	
Posture	Breast positioning	Measured angle	Classification	Measured angle	Classification	Measured angle	Classification
Trunk	MLO	6°	Acceptable	30°	Acceptable	9°	Conditionally acceptable
Arm flexion	MLO	71°	Not acceptable	74°	Not acceptable	69°	Not acceptable
Arm abduction	MLO	80°	Not acceptable	66°	Not acceptable	81°	Not acceptable
Head/neck	MLO	16°	Acceptable	4°	Acceptable	0°	Acceptable

The *postural alternatives* for the radiographer’s posture did not improve noticeably when the patient was positioned on the step (Fig. 4b and d).

The same tendency was observed when the radiographer and the patient were both seated. The angle of the trunk increased from 6 to 30°. The angle of arm in flexion and abduction kept the classification “not acceptable”.

### Radiographer taller than the patient—anthropometric combination 180 cm/153 cm

#### CC positioning

The radiographer assumed an orthostatic posture to position the breast. The trunk/spine was aligned with the mid-sagittal plane of the body. The right arm assumed a slight flexion and the forearm performed an internal rotation allowing patient positioning with the right hand. The left hand (not visible in the images) was used to position the breast, removing skin folds and helping on the breast compression. The right leg supported part of the radiographer’s body weight, while the left leg performed a slight flexion to reach the compressor foot pedal (Fig. 5a and b).

The angles of the trunk and neck/head were classified as “acceptable” during the breast positioning without any corrective measure. The right arm position was classified as “conditionally acceptable” (Table 7). When the *postural alternatives* take place the patient was placed on a step (Fig. 5b and d), and changes were identified mainly for the head/neck angles reducing from 32 to 18° (Table 7).

**Table 7:** Angles measured on the radiographer performing CC breast positioning (radiographer taller than the patient)

		Without intervention		Patient on a *step*	
Posture	Breast positioning	Measured angle	Classification	Measured angle	Classification
Trunk	CC	4°	Acceptable	3°	Acceptable
Arm flexion	CC	27°	Conditionally acceptable	33°	Conditionally acceptable
Arm abduction	CC	36°	Conditionally acceptable	49°	Conditionally acceptable
Head/neck	CC	32°	Acceptable	18°	Acceptable

#### MLO positioning

The radiographer performed severe trunk flexion. The head/neck segment was in hyperextension allowing the radiographer to see the breast while being positioned. The right arm was in flexion and abduction resting on the patient’s back. Both legs were flexed, the left leg was slightly flexed in a way to keep the body balanced and being able to reach the foot pedal at the same time. All the values obtained during the positioning without any correction were classified as “not acceptable” (Table 8).

**Table 8:** Angles measured on radiographer performing MLO breast positioning (radiographer taller than the patient)

		Without intervention		Radiographer seated		Patient on a *step*	
Posture	Breast positioning	Measured angle	Classification	Measured angle	Classification	Measured angle	Classification
Trunk	MLO	72°	Not acceptable	51°	Conditionally acceptable	14°	Acceptable
Arm flexion	MLO	139°	Not acceptable	128°	Not acceptable	60°	Conditionally acceptable
Arm abduction	MLO	120°	Not acceptable	95°	Not acceptable	99°	Not acceptable
Head/neck	MLO	−24°	Not acceptable	−18°	Not acceptable	0°	Acceptable

Positioning the patient on a step, as a *postural alternative* measure, the angles decrease to all of the anatomic areas considered in the postural evaluation of the radiographer. The angle of the trunk reduced from 72 to 51°, being classified as “conditionally acceptable”. The remaining angles regarding the other anatomical areas were classified as “not acceptable”.

The other *postural alternative* tested was seating the radiographer (Fig. 6b and d). In that situation, the angles were also reduced, improving in the trunk angle (from 72 to 14°) and in the head/neck angle (from −24 to 0°). This change in posture was enough to change the classification to “acceptable”. The position of the arm also improved during the flexion and was classified as “conditionally acceptable” while the abduction kept the “not acceptable” categorisation.

## Discussion

This study aimed to identify the most demanding postures for radiographers while performing mammography exams and to suggest *postural alternatives* to reduce the risk of WRMSDs based on evidence. In the previously performed study [1] about mammography activities, the repetition of the movements associated with breast positioning was shown. The risk of WRMSDs, mainly while performing the MLO projection, was verified. In the first phase of this study, similar results were found in all scenarios simulated. The most awkward postures were classified as “non-acceptable” according to the European standard (BS EN 1005–4: 2005).

*Postural alternatives* adjusted to the anthropometric characteristics of the radiographers and patients were simulated, in the second phase of the study, and improvements were observed. The most obvious were perceived in the most extreme situations, the radiographer taller or shorter than the patient.

In the second scenario simulated (radiographer shorter than patient) for breast CC positioning, *postural alternatives* with the radiographer on the step, a reduction of 35.9% and 40.0% on the angle of the arm flexed and abducted was observed. On the other hand, the angle of the trunk increased 14.3% and the angle of head/neck increased 19.0%. However, this increase did not raise the risk of WRMSDs as the classification of “acceptable” was kept. During the *postural alternatives* for MLO breast positioning, asking the patient to be on a step was enough to promote an improvement of radiographers’ posture. A reduction of 50.0% in the angle of the trunk and 100% in the flexion of the head/neck was verified. Nevertheless, the risk of WRMSDs still exists because the classification of the angles as “non-acceptable” was kept. For this specific situation, other strategies to improve the radiographers’ posture are necessary to prevent the occurrence of WRMSDs.

In the third situation (radiographer taller than patient), *postural alternatives* for positioning the patient on a step to perform breast CC views allowed also a reduction of 25.0% in the angle of the radiographer’s trunk and 43.8% in the angle of the neck/head. The angles of flexion and abduction of the arm were increased in 22.2% and 36.1% respectively but the classification of “conditionally acceptable” obtained without any intervention was preserved. For MLO position in the same *postural alternative* scenario, seating the radiographer, allowed a reduction of 100% of head/neck angle, 80.6% in the trunk angle, 56.8% and 17.5% in the angles of the arm during the flexion and abduction, respectively. These improvements in radiographers’ *postural alternatives* during mammography performance can reduce the risk of WRMSDs and for that reason should be implemented in a clinical context. Several authors [2, 4–6] showed in previous studies that the prevention should be prioritised, introducing changes that allow maintenance or even promotion of the workers’ health. WRMSDs sometimes are difficult to treat, being preferable to act at the level of the prevention, introducing protective equipment, hazard information, communication and right-to-know training, ergonomic consultation and assistance [4].

Other radiographer activities should be analysed, focused on the manipulation of other medical imaging devices and workstations but also evaluating the noise, temperature, impact of monitors in visual performance and patient handling. General studies have already analysed some of those topics but not targeting specifically the radiographers’ activities, health and safety promotion and prevention [2–12]. The analysis of the workflow and workload impacts should be also explored, mainly due to the introduction of new digital systems. The use of digital technologies brought new challenges and demands for radiographers’ activities as already shown in some studies [13–19]. The effects on radiographers’ health and safety needs to be identified, to prevent occupational disorders and the associated costs. The training and education has also a major role to help healthcare professionals be aware and apply safe strategies at work, as showed in other studies [2, 4, 6].

The main limitation of this study is related to the simulation of clinical practice. The images were not collected during mammography exam acquisitions to not disturb the patients and also to not affect the workload and workflow of mammography departments. The other limitation is related to the simulation of only three stature combinations. The methodology used in a previous study was followed, focusing mainly the extremes, as they were considered as the most challenging.

## Conclusions

The mammography equipment used in this study to simulate three different scenarios was not adjusted for the radiographers’ anthropomorphic characteristics. Performing CC and MLO mammography views can be highly demanding for radiographers, increasing the risk of WRMSDs. Postures classified as “not acceptable” were identified when standard positioning was performed. Introducing *postural alternatives* to the standard procedure (seating and/or raising patient or radiographer), for the most extreme scenarios such as the radiographer being taller or shorter than the patient, can reduce the risk of WRMSDs.
